# Weakening TPR-MBER correlation: Marker of success in India's malaria elimination progress

**DOI:** 10.6026/973206300221070

**Published:** 2026-02-28

**Authors:** Kapil Prajapat, Yeluri Sudeepa Libny, EA Syed Ashish, Pankaj Prasad, Alekhya Yalavarthi, Ankit Das, Kapil Chaudhary, Ishita Jain

**Affiliations:** 1 Department of Community & Family Medicine, All India Institute of Medical Sciences, Bhopal, Madhya Pradesh, India; 2 All India Institute of Medical Sciences, Bhopal, Madhya Pradesh, India; 3 Department of General Medicine, All India Institute of Medical Sciences, Bhopal, Madhya Pradesh, India; 4 Department of General Medicine, Mahatma Gandhi Memorial Medical College, Indore, Madhya Pradesh, India

**Keywords:** Malaria, Madhya Pradesh, monthly blood examination rate (MBER), test positivity rate (TPR), surveillance, NCVBDC, correlation

## Abstract

Malaria incidence in Madhya Pradesh has declined in recent years, reflecting strengthened surveillance and control measures. We analysed 2020-2024 Monthly Malaria Information System data to assess trends in transmission and the evolving relationship between the Monthly Blood Examination Rate (MBER) and Test Positivity Rate (TPR). A progressively weakening correlation between MBER and TPR was observed, indicating improved surveillance saturation and reduced underlying transmission. Thus, we show the potential utility of the MBER-TPR correlation as a dynamic indicator for monitoring progress toward malaria elimination.

## Background:

Malaria continues to be one of the world's deadliest vector-borne diseases, primarily caused by *Plasmodium falciparum* and Plasmodium vivax. Globally, in 2023, there were an estimated 263 million malaria cases and 597,000 deaths [1, 2]. Despite this high global burden, India has achieved a substantial decline in malaria incidence, as reported in the World Malaria Report 2024 [3]. India's achievements include marked reductions in malaria cases and deaths between 2017 and 2023. This progress is further evidenced by India's exit from the WHO's High Burden to High Impact group in 2024, marking a pivotal shift in its malaria elimination efforts [4]. Madhya Pradesh, India's second-largest state by area and centrally located, has strategic epidemiological importance in national malaria control. Historically, the state has contributed substantially to India's malaria burden. However, recent years have seen significant improvements, with Madhya Pradesh advancing from Category 3 to Category 1 under the National Framework for Malaria Elimination in 2023-a reflection of strengthened control indicators [5]. Nonetheless, ongoing monitoring of surveillance quality remains essential to sustain and accelerate progress toward malaria elimination in India. This study introduces a novel analytical approach: assessment of the temporal MBER-TPR correlation as a dynamic indicator of surveillance sensitivity. We hypothesise that, in high-burden or under-surveyed settings, increases in MBER positively correlate with TPR, reflecting enhanced case detection. Conversely, as surveillance coverage saturates and transmission intensity declines, this correlation weakens, signalling improvements in system performance. Therefore, it is of interest to analyse malaria trends in Madhya Pradesh from 2020 to 2024 and evaluate the MBER-TPR correlation. Madhya Pradesh was selected owing to its historical malaria burden, recent programmatic gains and availability of robust surveillance data.

## Materials and Methods:

This study was conducted in Madhya Pradesh (MP), a landlocked state in central India (surrounded by 7 states) covering 308,245 km^2^ and having a population of approximately 70 million (Census 2011). The state consists of 55 districts and experiences a tropical climate with distinct wet (June-September) and dry (October-May) seasons, with the monsoon period coinciding with peak malaria transmission. We selected MP as the study setting based on the following criteria: historical high malaria burden, recent programmatic improvements, geographic representativeness of central India and robust surveillance data availability. This study employed an ecological study design, which analyses population-level aggregated data rather than individual-level characteristics, suitable for studying time- based surveillance trends at the state level. Retrospective secondary malaria surveillance data collected under the National Center for Vector Borne Disease Control (NCVBDC) to examine population-level relationships between malaria testing intensity (MBER) and test positivity (TPR) in Madhya Pradesh. Monthly state-level malaria surveillance data were obtained from the official Monthly Malaria Information System (MMIS) available on the website of the NCVBDC, Ministry of Health and Family Welfare, Government of India. These reports provide standardised, validated and regularly updated data from all districts within Madhya Pradesh. All datasets used are in the public domain, ensuring transparency and reproducibility. The monthly data were extracted from January 2020 to December 2024 (60 months). The selected study period aligns with the timeline that includes India's intensified elimination efforts and the country's exit from the WHO's High Burden to High Impact (HBHI) group. The data variables included from NCVBDC's site were total positive malaria cases (TPC), blood examinations (BE) and confirmed cases of *Plasmodium falciparum* (Pf). To calculate the population-based indicator, i.e. MBER, annual population estimates for Madhya Pradesh were obtained from the Unique Identification Authority of India's (UIDAI) website for the years 2020 to 2024. We have included all the data (Universal sapling) for the above duration. MBER quantifies the proportion of the population tested for malaria and reflects diagnostic reach. Whereas the Test Positivity Rate (TPR), defined as the proportion of positive tests among all blood examinations conducted. These indicators were calculated monthly from January 2020 to December 2024, generating a total of 60 data points for each metric.

They were calculated as:

MBER = (BE in a Month/MP's Total population) *100

TPR = (TPC in a month/BE in the same month) *100

The raw data were reviewed for completeness and consistency. Missing values (sporadically occurring due to data gaps in NCVBDC reports) were addressed using a combination of forward (for months Oct-23 and Nov-23) and backward (For month Dec-23) imputation, assuming data stability within short time windows. This approach was used to preserve temporal continuity without introducing artificial trends. Descriptive statistics, including graphs, were used to explore trends in incidence, seasonal variations and species distribution.

## Novel analytical approach:

## Dynamic correlation analysis:

To evaluate surveillance performance over time, we investigated the correlation between MBER and TPR using Spearman's rank correlation coefficient (rho). Unlike static cross-sectional correlation, this study applied a sequential expanding time-window approach. The initial window covered January 2020 to June 2022. Each subsequent time window added one month, extending to include data up to December 2024. A total of 30 sequential correlation values (rho) were calculated. These rho values were then plotted monthly (from June 2022 to December 2024) to visualise how the strength and direction of the MBER-TPR Association evolved. This novel approach aimed to reflect shifts in surveillance effectiveness as diagnostic coverage expanded and malaria transmission declined. All analyses were performed using Microsoft Excel and JASP (Version 0.19.3, Apple Silicon compatible).

## Ethical considerations:

This study utilized anonymized, aggregated data from a public source, available through government portals. No patient identifiers or individual-level data were used; hence, institutional ethics approval was not required in accordance with national ethical guidelines for secondary data analysis. All data were handled securely and reported exclusively at the state level to ensure privacy protection.

## Results and Discussion:

**Figure 1:**
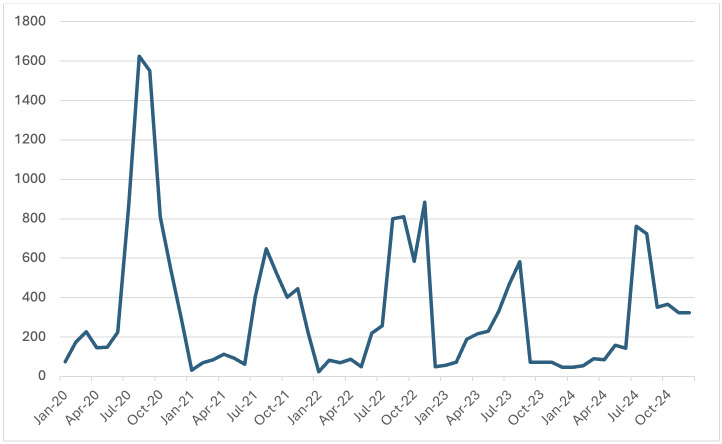
Total Positive Cases (TPC) of Malaria from 2020-2024 in MP

**Figure 2:**
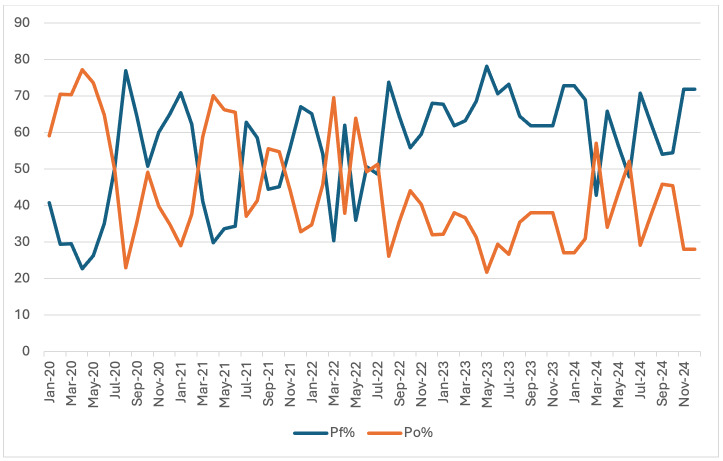
Cases caused by *P. falciparum* versus Other Plasmodium variants from 2020-2024 in Madhya Pradesh

This study offers important insights into malaria epidemiology and surveillance in Madhya Pradesh (MP), India, over a five-year period. The data show seasonal variation, a persistent burden of *Plasmodium falciparum* and evolving dynamics in diagnostic coverage and case detection. The correlation between the Monthly Blood Examination Rate (MBER) and Test Positivity Rate (TPR) provides a novel lens for evaluating the efficiency and sensitivity of malaria surveillance. Figure 1 shows a clear seasonal pattern in malaria incidence in Madhya Pradesh. Case numbers consistently peaked between July and November each year, aligning with the monsoon and post-monsoon periods. This pattern was especially prominent in 2020 and 2022, where the seasonal surge was more pronounced. The lowest malaria transmission occurred between January and May each year, indicating a consistent lull in the dry season. This seasonal variation underscores the influence of climatic and vector-related factors on transmission dynamics. This is consistent with national and regional studies that attribute heightened transmission to increased mosquito breeding due to water stagnation and humidity during this time. Studies conducted in India and globally support the finding that rainfall and temperature interact synergistically in malaria transmission [6,7- 8]. As shown in Figure 2, *Plasmodium falciparum* remained the dominant species, responsible for nearly 60% of all malaria cases throughout the study period. This species is responsible for the most severe malaria cases and is associated with higher case fatality rates [9, 10]. This trend persisted across both high- and low-transmission seasons, underscoring the epidemiological importance of *P. falciparum* in the region. The persistence of *P. falciparum* despite programmatic interventions underscores the need for continued vigilance, strict adherence to artemisinin-based combination therapy (ACT) protocols and resistance monitoring. Emerging data from the country have reported delayed parasite clearance in *P. falciparum*, highlighting the potential threat of resistance [11, 12].

**Figure 3:**
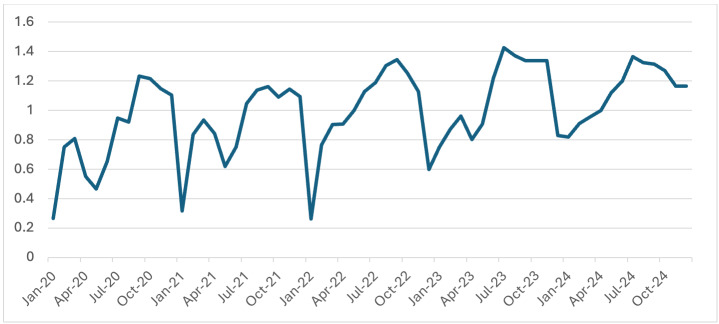
Monthly Blood Examination Rate (MBER) of Malaria from 2020-2024 in Madhya Pradesh

**Figure 4:**
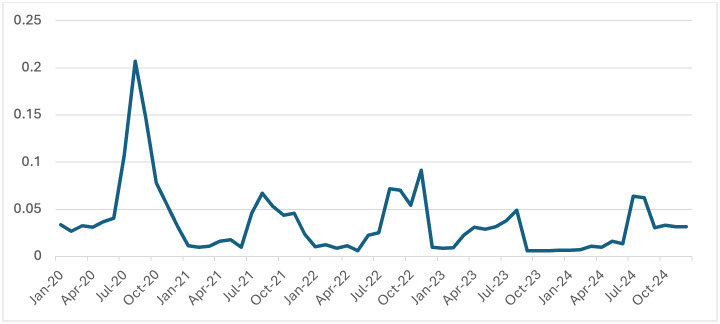
Test Positive Rate (TPR) of Malaria from 2020-2024 in MP

As depicted in Figure 3, the Monthly Blood Examination Rate (MBER) exhibited a steady upward trend from 2020 to 2024. In contrast, the Test Positivity Rate (TPR) showed a declining trend during the same period Figure 4. Initially, TPR was low between January and March 2020, followed by a sharp surge peaking in August 2020, when positivity exceeded 0.20- the highest value recorded during the study period. After this peak, TPR began to gradually decline, with most months from 2022 onwards showing positivity rates below 0.10. The increase in MBER over the study period suggests strengthened diagnostic outreach and improved responsiveness of the health system. MBER is recognized as a key surveillance metric in elimination settings, reflecting both health system engagement and diagnostic effort [13, 14]. The NCVBDC's expansion of microscopy training and deployment of rapid diagnostic tests (RDTs) has likely contributed to this positive trend. However, programmatic quality control in diagnostics remains critical for sustaining accuracy and reliability. In contrast to the increased MBER, TPR declined, indicating a reduction in malaria transmission. This trend is consistent with broader national data indicating a decline in slide positivity in many Indian states, following the implementation of intensified malaria control programs [15]. However, TPR should be interpreted in context: in high-testing settings, a low TPR may reflect true reduced burden, but may also signify inefficient testing if conducted in non-endemic or low-risk populations.

**Figure 5:**
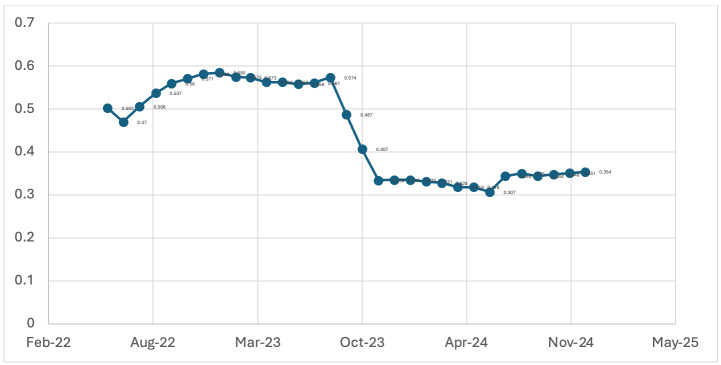
Sequential Spearman rho values showing weakening MBER-TPR association over time (2020-2024) Temporal Dynamics of the MBER-TPR Correlation

As shown in Figure 5, the correlation between MBER and TPR, measured using Spearman's rho, evolved significantly over time. Initially, from April 2023 until mid-2023, the correlation was moderately strong and relatively stable, with rho values ranging from approximately 0.55 to 0.59. This phase reflects a period during which enhanced testing efforts were effectively identifying malaria cases, suggesting efficient surveillance targeting. However, a marked decline in the correlation coefficient was observed in August 2023, indicating a potential shift in surveillance yield or underlying transmission patterns. From November 2023 through December 2024, the correlation stabilized at a lower range, with rho values between 0.33 and 0.36. The key novelty of this study lies in tracking the time-evolving correlation between MBER and TPR. The observed weakening of the correlation between MBER and TPR over the study period is the most significant finding, as it reflects Madhya Pradesh's successful transition from a high- burden state to one approach malaria elimination. In the initial phase, a strong positive correlation is expected; intensifying testing efforts (increasing MBER) effectively detects a high prevalence of infections, thus raising the TPR. However, as control measures take effect and true transmission declines, this relationship inevitably weakens. This signals the march of surveillance towards a saturation level for Malaria screening. Therefore, this weakening correlation is an indicator of the success of surveillance. Since a significant proportion of malaria testing in MP continues to rely on RDTs, low-parasitemia infections may remain undetected, potentially underestimating true transmission. Strengthening microscopy quality assurance in hotspot districts is crucial for accurate case detection in the elimination phase [16] his study is not without limitations. Based on aggregated secondary data, the study lacks granularity to assess sub district-level heterogeneities. Missing data imputation and reliance on UIDAI population estimates may introduce bias.

## Conclusion:

We show that case numbers consistently peaked between July and November each year, aligning with the monsoon and post-monsoon periods. The progressive weakening of the MBER-TPR correlation indicates successful reduction of transmission and surveillance saturation in Madhya Pradesh. This trend indicates that Madhya Pradesh has made significant strides in controlling malaria, as evidenced by a consistent decline in test positivity rates amid strengthened diagnostic efforts. Embracing this new surveillance tool, i.e., MBER-TPR correlation will surely help sustain momentum and achieve a malaria-free India.

## Source(s) of support and funding:

None
